# Comparison of Polyphenolic Content and Bioactivities Between Extracts from the Living Plants and Beach Deposits of the Submerged Brackish Water Angiosperm *Ruppia maritima*

**DOI:** 10.3390/molecules30132800

**Published:** 2025-06-29

**Authors:** Alkistis Kevrekidou, Nikolaos Goutzourelas, Stavroula Savvidi, Varvara Trachana, Andreana N. Assimopoulou, Ming Liu, Paraskevi Malea, Dimitrios Stagos

**Affiliations:** 1 Laboratory of Organic Chemistry, School of Chemical Engineering, Aristotle University of Thessaloniki, 54124 Thessaloniki, Greece or akevrek@cheng.auth.gr (A.K.); adreana@gapps.auth.gr (A.N.A.); 2 Department of Biochemistry and Biotechnology, School of Health Sciences, University of Thessaly, Biopolis, 41500 Larissa, Greece; nikgkoutz@gmail.com (N.G.); ssavvidi@uth.gr (S.S.); 3 Environmental Engineering Laboratory, Department of Chemical Engineering, Aristotle University of Thessaloniki, 54124 Thessaloniki, Greece; 4 Department of Biology, Faculty of Medicine, University of Thessaly, Biopolis, 41500 Larissa, Greece; vtrachana@med.uth.gr; 5 Natural Products Research Centre of Excellence-AUTH (NatPro-AUTH), Center for Interdisciplinary Research and Innovation (CIRI-AUTH), 57001 Thessaloniki, Greece; 6 Key Laboratory of Marine Drugs, Ministry of Education, School of Medicine and Pharmacy, Ocean University of China, Qingdao 266003, China; lmouc@ouc.edu.cn; 7 Laboratory for Marine Drugs and Bioproducts, Qingdao Marine Science and Technology Center, Qingdao 266237, China; 8 Department of Botany, School of Biology, Aristotle University of Thessaloniki, 54124 Thessaloniki, Greece

**Keywords:** *Ruppia maritima*, brackish water angiosperm, seagrass, antioxidant, anticancer, polyphenols, beach deposits, necromass

## Abstract

Bioactive extracts from living plants (LR) and beach deposits (NR) of the submerged brackish water angiosperm *Ruppia maritima* were examined for their antioxidant activity and anticancer potential. LR extract scavenged effectively free radicals with IC_50_ values of 38.00 μg/mL (DPPH^•^), 12.00 μg/mL (ABTS^•+^), 281.00 μg/mL (OH^•^), and 53.00 μg/mL (O_2_^•^), and exhibited reducing activity with an RP_0.5AU_ value of 37.00 μg/mL. NR extract retained a significant part of LR extract’s antioxidant activity by scavenging free radicals with IC_50_ values of 180.00 μg/mL (DPPH^•^), 60.00 μg/mL (ABTS^•+^), and 164.00 μg/mL (O_2_^•^), and exhibited reducing activity with an RP_0.5AU_ value of 107.00 μg/mL. Importantly, NR extract (IC_50_ value: 60.00 μg/mL) exhibited much higher inhibitory activity than LR extract (IC_50_ value: 1100.00 μg/mL) in XTT assay. HPLC analysis revealed that both *R. maritima* extracts contained phenolics, such as chicoric acid, quercetin-3-*O*-glucopyranoside, *p*-coumaric acid, 3,5-dimethoxy-4-hydroxicinnanic acid, *trans*-ferulic acid, and rutin hydrate, possessing antioxidant and/or anticancer activity. Thus, the present study showed for the first time that *R. maritima* extracts from either LR or NR are a promising source of bioactive compounds having beneficial properties for human health.

## 1. Introduction

Marine angiosperms (also known as seagrasses) and submerged brackish water angiosperms exhibit a dominant role in the structure and function of coastal and transitional ecosystems [1,2,3]. Submerged brackish water angiosperms, such as those of the genus *Ruppia* (family Ruppiaceae), tolerate a wide range of salinity values, and so they are entirely restricted at low intertidal elevations to permanent and ephemeral brackish lagoons, salt marshes, and sheltered shallow bays [2,3,4]. *Ruppia maritima* Linnaeus is the most prevalent brackish water angiosperm species of *Ruppia* genus [5]. *R. maritima* is a common species in European coastal lagoons, especially Mediterranean [4,6,7,8,9].

Since ancient times, seagrasses were used as food and in traditional medicine to treat skin diseases, fever, muscle and stomach pain, insect bites, and burns, as well as aphrodisiac, vasoprotective, anti-inflammatory, antioxidant, and contraceptive agents [10,11,12]. These activities of seagrasses are attributed to both their primary bioactive components (e.g., fibers, proteins, sulfated polysaccharides, acids, fats, and oils) [13,14], and secondary metabolites (e.g., polyphenols, alkaloids, and steroids) [11,15,16]. Possible bioactive compounds have been isolated from *R. maritima* [17], but their activities have not been fully investigated.

Seagrasses, and specifically submerged brackish water angiosperms, like *R. maritima*, are exposed to various biotic, abiotic, and anthropogenic stresses, due to the excessive diversity and complexity of their ecosystems [4,18,19,20], which may lead to reactive oxygen species (ROS) overproduction [16,21,22]. Subsequently, ROS overproduction stimulates enzymatic and non-enzymatic antioxidant molecules in plant cells [18,21,23,24,25,26]. (Poly)phenolic compounds possessing antioxidant activity, such as chicoric acid and flavonoid derivatives of quercetin and isorhamnetin, have been identified in *R. maritima* [17]. However, its extracts’ effects against free radical scavenging have never been assessed previously. Bioactive compounds from seagrasses have also exhibited anticancer activity [16,27,28]. However, extracts from *R. maritima* have not, so far, been investigated for their anticancer properties.

It is worth noting that not only the extracts from living plants, but also those from their beach deposits consisting of decomposing plants (e.g., *P. oceanica*, *Cymodocea nodosa*) that wash up on shores, contain minerals, (poly)phenols, carotenoids, tannins, active carbohydrate enzymes, lignocellulose degradation enzymes, and other natural derivatives, with antioxidant, antiviral, antifungal, antidiabetic, vasoprotective, and anticancer properties [16,29]. From ancient Egypt until today, seagrass beach deposits have been used in medicine to treat different pathological conditions such as pain, skin diseases, acne, hypertension, and inflammation [30]. Seagrasses have high resistance to decay [31] and their bioactive compounds (e.g., phenolics) are preserved during their deposition on shores [32].

Seagrasses and submerged brackish water angiosperms, due to their significant ecological importance, are protected by international conventions [3,33,34], and thus there is a great need to protect and preserve their habitats [35]. In Mediterranean countries, there is still no legislative framework that strictly prevents the removal of seagrass beach deposits from shores [16]. However, at the initiative of the municipal authorities, dead plant material of seagrasses (mainly of *P. oceanica* and *C. nodosa*) is often removed from coasts, unplanned and at high cost, and disposed of in landfills as waste. Thus, the possibility of utilizing marine and brackish water angiosperms’ beach deposits to produce high added value products possessing beneficial properties for human health, such as antioxidant or anticancer properties, is of great importance. Since, it may also lead to their proper utilization and management.

Thus, the aim of the present study was to prepare extracts from living plants (LR) and beach deposits on shores, also referred to as ‘necromass’ (NR), of the submerged brackish water angiosperm *R. maritima,* and identify their (poly)phenolic compounds. In addition, we used the collection area of *R. maritima* in ‘Monolimni’ Lagoon (Evros River Delta) as a ’case study’ to estimate the total quantity of phenolic compounds that can be produced from NR. Apart from phenolics, we compared the protein content between LR and NR plant material as an indication of the degree of NR decomposition. Moreover, we assessed the extracts’ free radical scavenging activity against 2,2-diphenyl-picrylhydrazyl (DPPH^•^), 2,2-azino-bis(3-ethylbenzthiazoline-6-sulfonic acid (ABTS^•+^), superoxide (O_2_^•−^), and hydroxyl (OH^•^) radicals, as well as their reducing activity. In addition, we evaluated ROS levels and the activity of the antioxidant enzymes, superoxide dismutase (SOD)-like and ascorbic peroxidase (APX)-like, in both LR and NR plant materials to determine whether these measurements could correlate with the tested extracts’ antioxidant activity. Furthermore, we examined LR and NR extracts for their ability to inhibit the growth of human colon cancer cells. To date, no other study has examined extracts from either LR or NR of *R. maritima* for their bioactivities.

## 2. Results and Discussion

### 2.1. Oxidative Stress, Antioxidant Enzyme Activity, and Protein Content of Ruppia maritima Living Plant (LR) and Beach Deposit (NR) Samples

The excessive diversity of seagrasses and submerged brackish water angiosperms leads to exposure to various biotic, abiotic, and anthropogenic stresses, and thus to ROS overproduction and stimulation of enzymatic and non-enzymatic antioxidant molecules [19,24,25,26,36]. As a result, oxidative stress contributes to the activation of antioxidant mechanisms, which neutralize or prevent the production of free radicals, and repair the affected molecules [16]. Thus, we assessed the hydrogen peroxide (H_2_O_2_) production and activity of the antioxidant enzymes SOD-like and APX-like in both LR and NR leaf samples of *R. maritima*, as an initial indication of the plant’s antioxidant potential. Moreover, the protein content was compared between the two samples to determine the degree of NR degradation.

**Figure 1:**
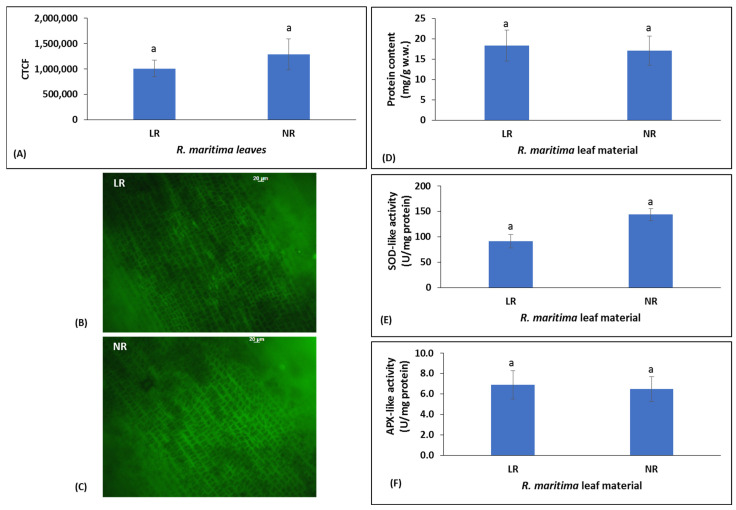
(**A**) Intracellular H_2_O_2_ levels in *Ruppia maritima* LR and NR leaf samples expressed as CTCF values. Fluorescence imaging of intracellular H_2_O_2_ production, obtained by immunofluorescent microscope after staining of (**B**) LR and (**C**) NR leaf samples with dichlorofluorescein diacetate (DCF-DA). Scale bar: 20 μm. (**D**) total protein content (mg/g wet weight) in the LR and NR leaf samples. Activity of the antioxidant enzymes (**E**) SOD-like and (**F**) APX-like. Values are the mean ± standard error (S.E.). Different lowercase letters denote significantly different values between the two extracts (*p* < 0.05).

Specifically, the assessment of the intracellular H_2_O_2_ levels expressed as the value of the corrected total cell fluorescence (CTCF), was used as an indicator of oxidative stress [24,25,26]. The results showed that the CTCF values of LR and NR leaf samples of *R. maritima* did not differ significantly (*p* > 0.05) and were 1,009,977 and 1,292,208, respectively (Figure 1D–F). Thus, oxidative stress existed in *R. maritima* LR and it slightly changed after plant abscission (i.e., in NR samples). Like H_2_O_2_ levels, there were no statistically significant differences (*p* > 0.05) in the activity of SOD-like and APX-like between LR and NR samples (Figure 1A,B). In particular, SOD-like activity was 91.89 and 144.10 U/mg of protein in LR and NR samples, respectively (Figure 1A), while APX-like activity was 6.93 and 6.58 U/mg of protein in LR and NR samples, respectively (Figure 1B). Thus, as expected, since oxidative stress did not differ between LR and NR samples, their antioxidant response was also similar. Moreover, protein content in the LR sample (18.33 mg/g wet weight) was not significantly different (*p* > 0.05) from that of the NR sample (17.09 mg/g wet weight) (Figure 1C), indicating that there was not significant decomposition of the NR sample until the collection time. This observation was consistent with other studies demonstrating that marine angiosperms are highly resistant to decay [31].

### 2.2. Determination of (Poly)phenolic Composition of LR and NR Extracts from Ruppia maritima

**Figure 2:**
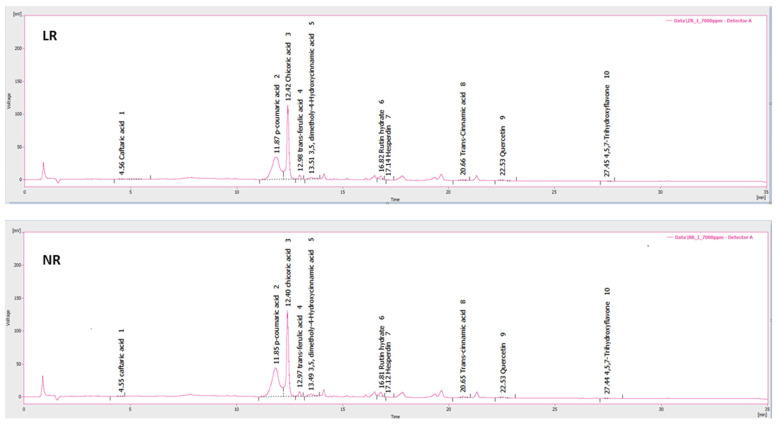
UHPLC-DAD chromatograms at 280 nm of the methanolic extracts from living plants (LR) and beach deposits (NR) of *Ruppia maritima*.

As a response to oxidative stress, seagrasses produce secondary metabolites (e.g., phenolic compounds), which, in turn, contribute to the bioactivities of the produced extracts [14,16,37]. Thus, we performed quantitative and qualitative analyses of phenolic compounds in both LR and NR extracts from *R. maritima* (Figure 2). From the 12 individual phenolic compounds analyzed, both LR and NR extracts contained the following compounds, in decreasing order regarding their amount: chicoric acid, quercetin-3-*O*-glucopyranoside, *p*-coumaric acid, rutin hydrate, sinapic acid (i.e., 3,5-dimethoxy-4-hydroxycinnamic acid), *trans*-ferulic acid, *trans*-cinnamic acid, and caftaric acid (Table 1; Figure 2).

The quantity of the individual phenolic compounds as mg/g of d.w. was similar between LR and NR extracts. Moreover, taking into account their extraction yield (i.e., 28% for LR and 10.3% for NR extract) (Table 1), the total phenolic compounds determined as mg/g of d.w. of the initial plant samples were only 2.4-fold higher in the LR sample compared to the NR sample (Table 1). In *P. oceanica* leaves, a significant loss of the total phenolic content (from 100- to 120-fold) and in the number of the individual phenolic compounds has been observed during its deposition on coasts [16,38]. Obviously, this occurred in *P. oceanica* due to the long period that the dead leaves remained on the shore [16,38]. Thus, the relatively low loss of phenolic compounds, from *R. maritima* NR samples compared to LR samples, may be attributed to the likely short time (about 48 h) that the former were remained on the shore before their collection. Other studies have also exhibited that seagrasses’ phenolic compounds are preserved after their deposition on shores [31]. Phenolics are not easily biodegradable because they are toxic to most microorganisms and therefore exhibit antimicrobial activity [39]. However, the phenolic compounds found in seagrass beach deposits may be affected by several biotic factors (e.g., microbial degradation) and abiotic factors (e.g., UV sunlight and temperature) [40,41,42]. Specifically, under aerobic conditions, microbial degradation of phenolics occurs through their oxygenation into catechols as metabolic intermediates, followed by ring cleavage at either the ortho or meta position [40]. Under anaerobic conditions, phenolics’ biodegradation proceeds through carboxylation, followed by dehydroxylation and dearomatization [40]. Common bacteria and yeasts capable of efficiently decomposing phenolic compounds include strains of *Pseudomonas*, *Bacillus*, *Rhodococcus*, and *Candida* [39,43]. In general, due to the limited number of studies on seagrass-associated microbiomes, the interplay between seagrasses and their microbiomes in the biosynthesis of bioactive compounds requires further investigation [44]. In addition, the extent of phenolic compound degradation depends on reactions with hydroxyl or superoxide ion radicals, and is influenced by sunlight (i.e., UV and visible light) and other environmental conditions (e.g., pH) [42]. Another factor influencing the chemical composition of seagrasses is the desiccation process [45,46], although this requires further investigation for *R. maritima*. It should be noted, however, that intertidal macrophytes including *Ruppia* species generally have a higher tolerance to desiccation and thermal stress than other macrophyte species [45]. Moreover, in our study, *R. maritima* NR was exposed to temperature and UV/solar radiation conditions likely similar to those experienced by the living plants. The lagoon was very shallow (i.e., 50–70 cm), and thus a large part of the living plants floated at the water surface and was directly exposed to the air.

Chicoric acid was the most abundant phenolic in both LR and NR extracts, with values of 41.09 and 45.4 mg/g d.w. of extract, respectively. In a previous study, chicoric acid was also reported as the most abundant phenolic compound of extracts from *R. maritima* and *R. cirrhosa* [17]. Specifically, the amount of chicoric acid was close to our values and ranged from 27.9 to 30.2 mg/g of d.w. in *R. maritima* extracts, and from 10.6 to 29.2 mg/g of d.w. in *R. cirrhosa* extracts [17]. It is noteworthy that chicoric acid is considered a high value-added nutraceutical, due to its beneficial properties for human health [47]. Specifically, chicoric acid has been reported to exhibit antioxidant [48], anti-inflammatory [49], antiviral [50], and anti-aging [51] properties, as well as activity against gastrointestinal diseases [49]. Moreover, it may regulate glucose and lipid metabolism [52], thus possibly exerting prevention against diabetes mellitus and cardiovascular diseases. However, the availability of chicoric acid is limited, and so there is the need to discover new sources of it [53]. Moreover, DellaGreca et al. [54] detected in *R. maritima* plants some other phenolic compounds, which were not determined in the present study. In extracts from *Cymodocea nodosa* and *P. oceanica* collected from the Mediterranean Sea, chicoric acid was also found in high amounts ranging from 3.19 to 18.52 and from 0.004 to 12.78 mg/g d.w. of extract, respectively [14,16,38,55,56,57]. It should be noted that all of the above phenolics identified in our *R. maritima* LR and NR extracts have also been found in extracts from the living leaves of *P. oceanica* seagrass [16,56,57]. However, only chicoric acid, *p*-coumaric acid, *trans*-cinnamic acid, *trans*-ferulic acid, and quercetin-3-*O*-glucopyranoside were found in extracts from *P. oceanica’s* beach casts [16,38,57]. In extracts from living plants of *C. nodosa*, among the above substances, chicoric acid, caftaric acid, *p*-coumaric acid, quercetin-3-*O*-glucopyranoside, and *trans*-cinnamic acid were detected, whereas in its beach deposit extracts, only chicoric acid remained [10,14,58].

The phenolic profile of the above seagrass species is similar and relatively simple, with only one or two phenolic compounds dominating. This may be due to the fact that these organisms reproduce mainly through vegetative reproduction, thus limiting genetic inconsistencies [57]. Additionally, although seagrasses colonized the marine environment from terrestrial angiosperm ancestors, no fundamental modifications have appeared in their phenolic profiles [58].

### 2.3. Antioxidant Activity of LR and NR Extracts from Ruppia maritima

**Figure 3:**
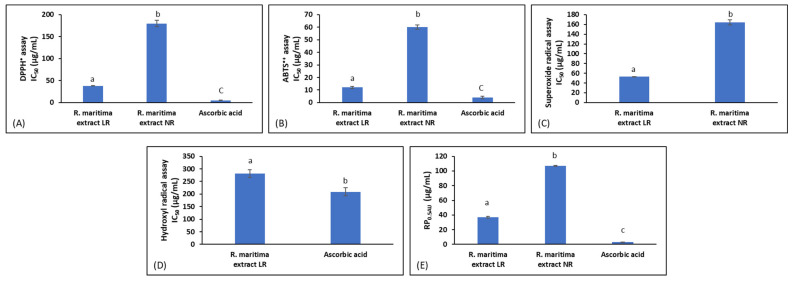
The IC_50_ values (μg/mL) of scavenging activity against (**A**) DPPH^•^, (**B**) ABTS^•+^, (**C**) hydroxyl (OH^•^), and (**D**) superoxide (O_2_^•−^) radicals, and (**E**) RP_0.05AU_ values of reducing power of the *Ruppia maritima* extracts from living plant (LR) and beach deposit (ΝR) samples. The NR extract did not exhibit an IC_50_ value in the hydroxyl radical assay at the tested concentrations (i.e., until 1000 μg/mL). Values are the mean ± SD of at least three independent experiments. Different lowercase letters denote significantly different values between the two extracts (*p* < 0.05).

*R. maritima* LR and NR extracts were examined in vitro for their antioxidant capacity. The results showed that both LR and NR extracts scavenged DPPH^•^ with IC_50_ values of 38.00 and 180.00 μg/mL, respectively (Figure 3). Moreover, both extracts scavenged ABTS^•+^ (IC_50_ values: LR 12.00 μg/mL vs. NR 60.00 μg/mL) and O_2_^•−^ (IC_50_ values: LR 53.00 μg/mL vs. NR 164.00 μg/mL) (Figure 3). Remarkably, in OH^•^ scavenging assay, only the LR extract exhibited an IC_50_ value, which was equal to 281.00 μg/mL (Figure 3). It was remarkable that both extracts exhibited more potency in ABTS^•+^ assay than DPPH^•^ assay, since their IC_50_ values were lower in the former assay. The ability of the tested extracts to scavenge O_2_^•−^ and OH^•^ is significant. Both of these free radicals are produced in living organisms through a number of biochemical and physiological processes, and their overproduction may lead to oxidative stress and subsequently to different diseases [59]. Ascorbic acid, the positive control of these assays, as a pure compound, had much lower IC_50_ values than the tested extracts (ascorbic acid could not be tested only in O_2_^•−^ assay because it can reduce NBT) [60] (Figure 3). However, in OH^•^ scavenging assay, LR extract had scavenging activity comparable to that of ascorbic acid (Figure 3).

In addition, we assessed the reducing activity of both extracts. As known, reducing capacity is an indicator of antioxidant activity, since free radicals are neutralized after receiving electrons [61]. Thus, in reducing power (RP) assay, LR and NR extracts exhibited RP_0.05AU_ values of 37.00 and 107.00 μg/mL, respectively (Figure 3).

The observed antioxidant capacity of the tested extracts could be attributed to their phenolic compounds identified by HPLC, since most of them have been reported to possess antioxidant activity. For example, several studies have demonstrated chicoric acid, the most abundant of the identified phenolics, to act as a free radical scavenger and protect cells from oxidative stress [62,63,64]. Quercetin-3-*O*-glucopyranoside has also been reported to scavenge ABTS^•+^ and DPPH^•^ radicals and possess reducing activity [65]. Spectroscopic analysis has also revealed that *p*-coumaric acid effectively scavenged ABTS^•+^ and DPPH^•^ radicals and exhibited reducing activity [66]. Furthermore, several cell culture [67] and in vivo studies [68] have shown *p*-coumaric acid to protect from ROS-induced harmful conditions. In addition, rutin hydrate has been demonstrated to scavenge, with high potency, ABTS^•+^ and DPPH^•^ radicals [69]. Nićiforović et al. [70] reported sinapic acid scavenged DPPH^•^ and O_2_^•−^ radicals, and protected different tissues, such as gastric [71], nervous [72], testicular [73] and renal [74] tissues, from oxidative stress-induced damage.

Since lower IC_50_ or RP_0.05AU_ values indicate higher antioxidant capacity, all the above antioxidant assays clearly demonstrated that the LR extract possessed stronger antioxidant activity than the NR extract. Specifically, the LR extract had from 2.9- to 5.0-fold lower IC_50_ values than the NR extract in the antioxidant assays. As a rule of thumb, in in vitro antioxidant assays such as those used in the present study, differences in IC_50_ greater than 2-fold are generally considered biologically meaningful in the literature. However, the IC_50_ values of the NR extract indicated that the beach deposits of *R. maritima* retained a significant part of the antioxidant potency of the living plant. Obviously, differences in chemical composition between LR and NR extracts accounted for their different antioxidant activity. However, our results did not show great changes in the (poly)phenolic composition between the two extracts. Thus, the present results suggested that not only the tested phenolics, but also other bioactive compounds, contribute to the antioxidant activity of *R. maritima* extracts. Moreover, there was no significant difference in either oxidative stress (i.e., H_2_O_2_ production) or antioxidant enzyme levels of *R. maritima* LR and NR samples before they were used for extraction. However, since LR and NR extracts exhibited different antioxidant activity, H_2_O_2_, SOD-like, and APX-like levels cannot be suggested as reliable indicators of the antioxidant capacity of the produced extracts.

Interestingly, the present study is the first to examine the antioxidant activity of extracts from either living plants or beach deposits of *R. maritima*. Enerstvedt et al. [17] isolated a crude extract from living leaves of *R. cirrhosa* using a method similar to ours. However, they also prepared a purified extract by applying the crude extract to a resin column. The crude extract of *R. cirrhosa* had an IC_50_ value of 152.9 μg/mL in DPPH^•^ assay, while the purified extract had a value of 31.8 μg/mL [17]. The antioxidant potency of the purified extract of *R. cirrhosa* was close to that of our *R. maritima* LR extract. Furthermore, the fact that the *R. cirrhosa* crude extract had antioxidant potency similar to that of our NR extract provided further evidence that the beach deposits of *R. maritima* retained significant antioxidant activity. Moreover, in one of our previous studies [16], extracts from beach cast leaves of *P. oceanica* exhibited much lower antioxidant activity compared to the *R. maritima* NR extract. For example, in DPPH^•^ and ABTS^•+^ assays, extracts from beach-cast leaves of *P. oceanica* had IC_50_ values of 2850 and 660 μg/mL, respectively [16].

### 2.4. Inhibitory Activity of LR and NR Extracts from Ruppia maritima Against Cancer Cell Growth

**Figure 4:**
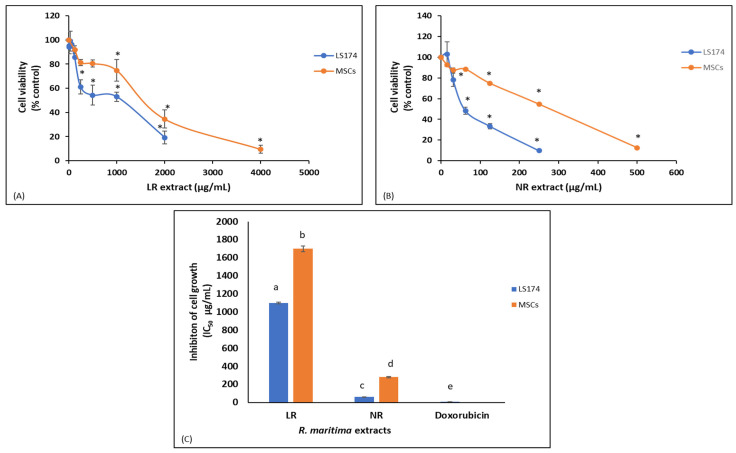
Dose-dependent inhibition of (**A**) living plant (LR) and (**B**) beach deposit (NR) extracts against growth in colon cancer cells (LS174) and mesenchymal stem cells (MSCs). (**C**) IC_50_ values (μg/mL) of *Ruppia maritima* LR and NR extracts against LS174 and MSC growth. Values are the mean ± SD of at least three independent experiments. Different lowercase letters denote significantly different values between the two extracts (*p* < 0.05). (*) Statistically significant difference from control (i.e., untreated) cells (*p* < 0.05).

In addition, we examined the LR and NR extracts from *R. maritima* for their inhibitory activity against colon cancer cell growth. The results showed that both extracts dose-dependently inhibited human colon cancer LS174 cell viability (Figure 4). Specifically, the LR extract exhibited a significant reduction in cell viability, by 39% at 250.00 μg/mL, while at 2000.00 μg/mL the cell viability was reduced by 80% (Figure 4). The NR extract demonstrated a significant decrease in cell viability, by 22% at 30 μg/mL, while at 250 μg/mL the reduction in cell viability was 90% (Figure 4). The IC_50_ values of LR and NR extracts against colon cancer cell growth were 1100.00 and 60.00 μg/mL, respectively (Figure 4). The IC_50_ value of the positive control, doxorubicin, was 9.13 μg/mL (Figure 4). Intriguingly, while the LR extract was more potent than the NR extract in all antioxidant assays, the latter exhibited about 18-fold higher inhibitory activity against cancer cell growth than the former. In cell proliferation assays, such as XTT assay, differences in IC_50_ values greater than 2-fold are considered biologically meaningful. This result suggested that the *R. maritima* extracts’ bioactive compounds accounting for the inhibition of cancer cell growth were different from those responsible for the antioxidant activity.

To examine the selective cytotoxic activity of the tested extracts against cancer cells, their cytotoxicity was also assessed against normal cells, specifically human mesenchymal stem cells (MSCs). The results showed that the LR extract had a significantly higher IC_50_ value in MSCs than LS174 cells (IC_50_: 1700.00 vs. 1100.00 μg/mL, respectively; Figure 4). Similarly, the NR extract exhibited a significantly higher IC_50_ value in MSCs than in LS174 cells (IC_50_: 280.00 vs. 60.00 μg/mL, respectively; Figure 4). Specifically, the selectivity index (SI), (i.e., the ratio of the IC_50_ in cancer cells to the IC_50_ in normal cells) was 1.5 for the LR extract and 4.7 for the NR extract. An SI greater than 2 is generally considered indicative of significant selectivity in cancer cell inhibition [75]. Thus, the present results indicated that, the NR extract in particular may exert selective inhibitory activity against colon cancer cell growth.

Among the identified phenolics, chicoric acid was the most abundant in *R. maritima* extracts. Plant extracts rich in chicoric acid have been demonstrated to possess anticancer properties [76,77]. For example, extracts derived from the terrestrial plants *Linum numidicum*, *Linum trigynum*, and *Taraxacum officinale*, which were rich in chicoric acid, inhibited the growth of liver, prostate, and breast cancer cell lines [76,77]. A number of studies have also shown that *p*-coumaric acid exerted cytotoxic effects on human epidermoid, melanoma, gastric, glioblastoma, and colorectal cancer cells [78,79,80,81,82]. Specifically, *p*-coumaric acid inhibited colon cancer cell growth through induction of mitochondrial-dependent apoptosis by downregulating the anti-apoptotic Bcl-xL and Bcl-2 proteins and upregulating the pro-apoptotic BAX protein. Moreover, *p*-coumaric acid caused cell cycle arrest by downregulating cdc2/cyclin B activity [83]. In addition, sinapic acid has been shown to inhibit human prostate, pancreatic, and colon cancer cell lines [84,85,86]. Regarding human colon cancer cells, sinapic acid induced apoptosis through an increase in the pro-apoptotic cleaved caspase-3 and BAX proteins [86].

However, the quantities of the above phenolics did not change significantly between the LR and NR extracts, while the NR extract exhibited higher inhibition of cancer cell growth. Thus, apart from the identified phenolics, other NR extract’s bioactive compounds may have also attributed to the cytotoxicity against cancer cells. The processes of *R. maritima* leaf abscission and subsequent drying, as well as the existence of distinct degradation metabolites, might lead to the production of metabolites having cytotoxic properties against cancer cells. Interestingly, microbial metabolites of polyphenols have been reported to enhance their bioactivities [44].

### 2.5. Case Study for the Quantification of the Bioactive Extract Produced by Ruppia maritima Beach Deposits in the Collection Area

Submerged brackish water angiosperms, such as *R. maritima*, in coastal and transitional habitats, are of significant ecological importance. Moreover, the present findings indicated that extracts from living *R. maritima* plants contain significant amounts of (poly)phenolic compounds (Table 1). Thus, living *R. maritima* plants can be utilized to produce extracts exhibiting beneficial bioactivities for human health [87]. For example, since *R. maritima*’s polyphenolic extracts exhibited antioxidant activity, they could be used as food supplements or incorporated into functional foods to protect from oxidative stress [87,88]. In addition, *R. maritima* extracts, due to their antioxidant activity, could be used in cosmetic products aimed at protecting from UV-induced oxidative stress, which leads to skin aging [89]. Moreover, seagrass extracts with antioxidant activity and rich in chicoric acid, such as those from *R. maritima*, have been shown to maintain skin elasticity through promotion of collagen synthesis, and to prevent signs of aging [89]. Finally, *R. maritima* extracts may be used to develop nutraceuticals for treating oxidative stress-induced diseases and prevention from cancer [90]. Natural compounds are often renewable resources and tend to exhibit lower toxicity and fewer side effects compared to conventional drugs [90]. Since these extracts are suggested for product development, it is worth noting that they retained their tested bioactivities after three years of storage at −20 °C. Of course, further studies are needed to investigate, for example, the bioavailability or potential adverse effects of the tested extracts. So far, available data on these issues come from the literature and concern their main identified compounds (i.e., chicoric acid, quercetin-3-*O*-glucopyranoside, and p-coumaric acid). Thus, pharmacokinetic studies on chicoric acid have demonstrated its bioavailability to be approximately 1.5% after oral administration of extracts containing 2% of chicoric acid in rats [91]. Chicoric acid’s bioavailability may be enhanced by encapsulation in nanomicelle formulations [92]. Quercetin-3-*O*-glucoside is absorbed by humans via specific transporters in the small intestine [93]. *p*-Coumaric acid exhibits efficient absorption and metabolism in animal models, with preliminary evidence suggesting similar bioavailability in humans [94,95]. Chicoric acid, quercetin-3-*O*-glucopyranoside, and *p*-coumaric acid are generally considered safe for human consumption. However, like any bioactive compound, they may have potential adverse effects such as allergies, gastrointestinal disturbances, nephrotoxicity, and drug interactions, particularly at high doses or in sensitive individuals [53,96,97,98].

Moreover, there should be acceptable management programs exerting the least possible impact on *R. maritima* ecosystems. Another option could be the development of cultivation methods for *R. maritima*. Moreover, the present results suggested that *R. maritima* beach deposits, after taking into account acceptable management programs, could be used for (poly)phenolic compounds’ production, since significant amounts of these compounds remain after plants’ deposition on coasts.

The collection area of the Monolimni Lagoon, located in the estuary of the Evros River (northeastern Aegean Sea, Greece), was used as a ‘case study’ to estimate the total amount of NR extract and the individual phenolic compounds that can be produced. In this area, along a 100 m shoreline, the volume of *R. maritima* beach deposits was calculated to be about 7.0 m^3^. Thus, *R. maritima* beach deposits were calculated to be 94.28 kg Kg wet weight (or 56.39 kg d.w., after drying at 50 °C). Given that the NR extraction yield was 10.3% (Table 1), the amount of NR extract that could be produced corresponds to 5.81 kg, containing a total of 438.71 g d.w. of the seven phenolic compounds quantified by HPLC (Table 2). The amount of the individual phenolic compounds that can be produced in this area is presented in Table 2. Currently, to the best of our knowledge, there are no management regulations for *R. maritima* beach deposits. However, in management programs of seagrasses, such as those for *P. oceanica*, up to 20% of its beach deposits is usually allowed for collection [16]. If the management programs for *P. oceanica* were also applied for *R. maritima* beach deposits, the total amount of *R. maritima* NR extracts that could be used from the collection area would be 1.16 kg. Similarly, the individual phenolic compounds that could be used would correspond to 20% of their estimated production, as presented in Table 2.

## 3. Materials and Methods

### 3.1. Sampling of Living Plants and Beach Deposits of Ruppia maritima

**Figure 5:**
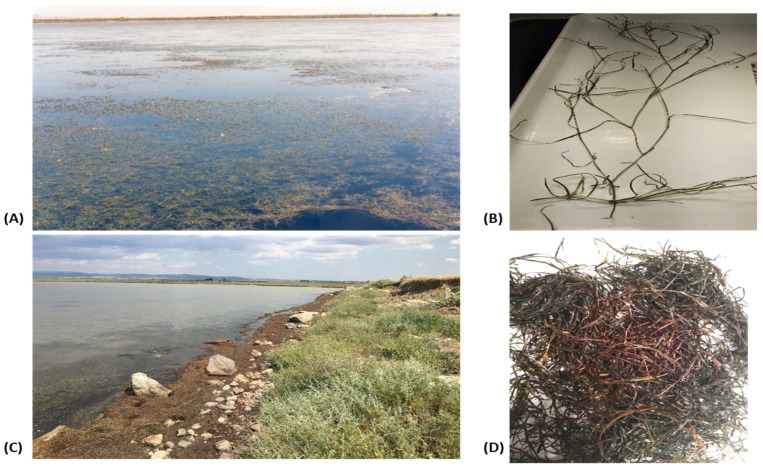
(**A**) View of the *Ruppia maritima* meadow at ‘Monolimni’ Lagoon at the estuary of the Evros River (northeastern Aegean Sea, Greece). (**B**) Macroscopic view of *R. maritima* living plants (LR). (**C**) Beach deposits or necromass (NR) of *R. maritima* on the shore of the collection area. (**D**) Macroscopic view of *R. maritima* beach deposit material.

We collected plants of the submerged brackish water angiosperm *R. maritima* Linnaeus in early July 2022 from a depth of 60 cm from the ‘Monolimni’ Lagoon (40°46′ N, 22°03′ E) of the Evros River Delta (northern Aegean Sea, Greece). The ‘Monolimni’ Lagoon occupies an area of approximately 1.12 km^2^, connects to the sea through a 15 m wide opening, and is permanently flooded. In the collection period, the composition of the vegetation in the inner part of this lagoon was homogeneous, consisting mainly of *R. maritima* plants (Figure 5A) and the macroalga *Gracilaria bursa-pastoris.* In this area, the perennial population of *R. maritima* grows from April to October and reproduces during summer, in the innermost part of the permanent Monolimni Lagoon [4]. Like other perennial *Ruppia* populations growing in the Mediterranean area, plant decomposition is observed after early to mid-summer, and then from early or mid-autumn onwards, leading to beach deposits on the shores [4].

We collected *R. maritima* living plant samples (LR) (Figure 5A,B) using a 20 cm diameter acrylic corer that penetrated the sediment to a depth of 30 cm [4]. The plant samples were then transported to the laboratory in containers with brackish water. The plant leaves used in our study presented the following phenological characteristics: leaf length 8.8 ± 0.3 cm, leaf width 0.98 ± 0.01 mm, number of leaves per shoot 2.67 ± 0.07, number of shoots/m^2^ 3500 ± 10, and leaf biomass 25.5 g d.w./m^2^. Moreover, the roots of the plants had the following phenological characteristics: orthotropic rhizome biomass 3.5 ± 1.8 g d.w./m^2^, plagiotropic rhizome biomass 8.6 ± 1.7 d.w./m^2^, root biomass 3.5 ± 0.4 g d.w./m^2^, orthotropic rhizome internode length 3.9 ± 0.2 cm, plagiotropic rhizome internode length 0.8 ± 0.07 cm, and root length 5.1 ± 0.3 cm.

Beach deposit NR material consisting of whole *R. maritima* plants washed up on the shore was also collected from the study area (Figure 5C,D). In July 2022, in the collection area of the *R. maritima* beach deposits, the volume and the total mass of NR material that washed up on the shores were estimated along a 100 m coastline. Field observations in the days preceding the sampling day and on the sampling day (7 July 2022) indicated that the plant deposits remained deposited for less than 48 h. During this period, the air temperature ranged between 18 °C and 31 °C (mean 26 °C) and the day length was approximately 14 h. The length of the collection area’s coastline was 100 m and the width (i.e., the distance between the lowest and highest point of the NR deposition on the coast) was 2 m (mean value) (Figure 5C). The height of the NR measured from the coast level was 4 cm (mean value) (Figure 5C).

In addition, ten samples of NR were collected using a metal frame of 40 × 40 cm. After collection, the samples were dried in an oven at 50 °C for two days. The drying temperature at 50 °C was chosen because, according to the literature, it does not appear to affect phenolic composition or their bioactivities [44,99]. For example, the ideal drying temperatures for plant material to retain polyphenols and flavonoids and their antioxidant activity have been reported to range from 55 to 70 °C [99]. Moreover, Ameen et al. [44] reported that the best extraction conditions for seagrasses are usually achieved by maintaining temperatures between room temperature and 60 °C. In addition, in the marine angiosperm *P. oceanica*, heat treatment at 60 °C for a short duration (i.e., two days) was found to be more effective for recovering phenolic compounds compared to five days at 37 °C [38]. After drying, the plant material’s weight was assessed. Based on the above measurements, we estimated the mass of the NR material along a 100 m coastline used as a ‘case study’ area. Finally, taking into account the extraction yield percentage of NR extract and the amount of each phenolic compound in it, the amount of the NR extract and each phenolic compound that could be produced in the ‘case study’ area were estimated.

### 3.2. Imaging of Hydrogen Peroxide Production in Leaves from Living Plants (LR) and Beach Deposits (NR) of Ruppia maritima

We assessed the H_2_O_2_ production in *R. maritima* living leaves (i.e., LR) of different ages (juvenile, intermediate, and adult leaves; *n* = 9) originating from 3 bundles, as well as in leaf material (*n* = 9 leaf pieces) from *R. maritima* beach deposits (i.e., NR), as described by Kevrekidou et al. [16] (see also Supplementary File S1). The samples were observed under a Zeiss AxioImager Z.2 fluorescence microscope equipped with an MRc5 Axiocam. The cell fluorescence intensity was calculated with Image J software version 1.54 software (U. S. National Institutes of Health, Bethesda, MD, USA). The corrected total cell fluorescence (CTCF) values were calculated according to the following equation:

CTCF = integrated density − (area of selected cell × mean fluorescence of background readings)(1)

Their mean values were calculated from 81 measurements for LR leaves, namely, three regions per leaf × three segments (apex, middle, base) per leaf × 9 leaves, and from 27 measurements for NR material (i.e., three regions per leaf piece × 9 leaf pieces).

### 3.3. Assessment of the Activity of the Antioxidant Enzymes SOD-like and APX-like and Protein Content in Leaves from Living Plants (LR) and Beach Deposits (NR) of Ruppia maritima

Three deep-freeze (−80 °C) leaf samples of both LR and NR materials were ground in liquid nitrogen. Each sample (100 mg wet weight) was treated in triplicate with 3 mL of 50 mM sodium phosphate buffer (pH 7.8) containing 0.1 mM of ethylenediaminetetraacetic acid (EDTA) and 2% *w*/*w* of polyvinylpolypyrrolidone (PVPP), then centrifuged at 16,500 × *g* for 30 min at 4 °C.

The protein content (mg/g w.w.) was measured spectrophotometrically using a PharmaSpec UV-1700 spectrophotometer (Shimadzu, Tokyo, Japan), according to the Bradford [100] method (see also Supplementary File S1).

SOD-like activity (U/mg protein) was measured, as proposed by Beyer and Fridovich [101] (see also Supplementary File S1), and APX-like activity (U/mg protein) was measured according to Nakano et al. [102] (see Supplementary File S1). The activity of both enzymes was determined using a PharmaSpec UV-1700 spectrophotometer (Shimadzu, Tokyo, Japan). The protein content of the samples was also used to normalize the values of SOD-like and APX-like activities.

### 3.4. Preparation of Ruppia maritima Extracts

We prepared the extracts from *R. maritima* living plants (LR) and beach deposits (NR), as previously described [16] (see also Supplementary File S1). The weight of the dried powder was measured to determine the percentage yield of the extraction process, using the following equation:

Extraction yield (%) = [dry extract (g)/dry seaweed (g)] × 100(2)

The extracts were kept at −20 °C until further use.

### 3.5. Assessment of Phenolic Coompounds in Ruppia maritima Extracts

The individual phenolic compounds in the LR and NR extracts were determined by UHPLC-DAD analysis as described previously [16] (see also Supplementary File S1). Identification and quantification of the individual phenolic compounds in all samples were based on the following standards: caftaric acid (purity > 97%, Sigma-Aldrich), chicoric acid (purity > 98%, Glentham, Corsham, UK), quercetin-3-*O*-glucopyranoside (purity > 99%, Extrasynthese, Genay Cedex, France), caffeic acid (purity 99%, J&K Scientific, Shanghai, China), (-)-epigallocatechin gallate (purity 95%, Thermo Fisher Scientific, Waltham, NE, USA), *p*-coumaric acid (purity 98%, Sigma Aldrich), *trans*-ferulic acid (purity 99%, Sigma Aldrich), sinapic acid (purity 98%, Sigma Aldrich), rutin hydrate (purity > 94%, Sigma Aldrich), *trans*-cinnamic acid (purity 99%, Sigma Aldrich), hesperidin (purity 95%, Sigma Aldrich), and 4′,5,7-trihydroxyflavone (purity 97%, Thermo Fisher Scientific, Waltham, NE, USA) (see Supplementary File S1 for details). Subsequently, the standards were diluted in methanol at concentrations ranging from 0.78 to 200.00 mg/L to construct calibration curves (Supplementary File S1), and analyzed at 280, 270, 328, and 318 nm. The phenolic content of the extracts was analyzed at 7.0 mg/mL in methanol. Moreover, considering the extraction yield (%) of each extract, the phenolic compounds were also quantified in mg/g of d.w. of the initial LR and NR materials.

### 3.6. Free Radical Scavenging Activity of Ruppia maritima Extracts

We evaluated the ability of the extracts to scavenge free radicals in vitro using DPPH^•^, ABTS^•+^, OH^•^, and O_2_^•−^ assays. All free radical scavenging assays were performed accordingly to one of our previous studies [103] and are described in detail in Supplementary File S1. In all assays, we evaluated the percentage of radical scavenging capacity (RSC) of the tested samples according to the following formula:

RSC (%) = [(A_control_ − A_sample_)/A_control_] × 100 (3)

where A_control_ and A_sample_ are the absorbance values of the control and the sample, respectively. The IC_50_ value, representing the concentration at which 50% of the free radical scavenging occurred, was calculated using a four-parameter logistic regression model with the ‘Quest Graph™ IC_50_ Calculator’ (AAT Bioquest, Inc., Pleasanton, CA, USA) [104]. Ascorbic acid was used as a positive control for the free radical scavenging activity. Each experiment was performed in triplicate and was repeated on at least three different occasions.

### 3.7. Reducing Power (RP) Activity of Ruppia maritima Extracts

Reducing power was assessed according to one of our previous studies [103] and is described in detail in Supplementary File S1. The RP_0.5AU_ value, indicating the extract concentration that caused an absorbance of 0.5 at 700 nm, was determined using a four-parameter logistic regression model with the ‘Quest Graph™ IC_50_ Calculator’ [104]. Ascorbic acid was used as a positive control. Each experiment was performed in triplicate and was repeated on at least three different occasions.

### 3.8. Cell Culture Conditions

The human colon LS174 cancer cell line was obtained from ATCC company (Manassas, VA, USA). Human MSCs were obtained from the Wharton Jelly of umbilical cords from term-gestation newborns after birth, with consent obtained from the parents in accordance with the principles of the Declaration of Helsinki, as previously described [105]. LS174 cancer cells were cultured in normal Dulbecco’s modified Eagle’s medium (DMEM, Gibko, Paisley, UK). DMEM contained 10% (*v*/*v*) fetal bovine serum, 100 units/mL of penicillin, and 100 units/mL of streptomycin (Gibko, Paisley, UK). MSCs were cultured in normal Dulbecco’s modified Eagle’s medium (DMEM), high-glucose with stable glutamine and sodium pyruvate (BioWest, Miami, FL, USA), plus 10% fetal bovine serum (FBS; Thermo Fisher Scientific, Waltham, MA, USA) and 1% penicillin/streptomycin (Thermo Fisher Scientific, Waltham, MA, USA). The cells were cultured in plastic disposable tissue culture flasks at 37 °C in 5% CO_2_.

### 3.9. XTT Assay for Assessing the Inhibitory Activity of Ruppia maritima Extracts Against Cancer Cell Proliferation

We assessed the inhibition of LS174 cancer cell proliferation using the XTT assay kit (Roche, Munich, Germany), which was performed according to one of our previous studies [103] (see also Supplementary File S1). For comparison purposes, we also tested extracts’ cytotoxic activity in normal human MSCs cells. The data were expressed as a percentage of inhibition using the following formula:

Inhibition (%) = [(O.D._control_ − O.D._sample_)/O.D._control_] × 100 (4)

where O.D._control_ and O.D._sample_ indicated the optical densities of the negative control and the tested extract, respectively. The IC_50_ value was calculated using a four-parameter logistic regression model with the ‘Quest Graph™ IC_50_ Calculator’ [104]. The anticancer drug doxorubicin was used as positive control. The experiment was conducted in triplicate and repeated at least three times.

### 3.10. Statistical Analysis

The results are expressed as mean ± standard error (mean ± S.E.) or mean ± standard deviation (mean ± S.D). For the free radical scavenging and XTT assays, the statistical analysis was based on one-way ANOVA followed by Dunnett’s T3 test for multiple pair-wise comparisons. For CTCF values, antioxidant enzyme activities, and protein content, the non-parametric Mann–Whitney U-test was applied to determine the significant differences in the measured variables, as the primary analysis on both raw and log-transformed data indicated unequal variances. Differences were considered significant at *p* < 0.05. SPSS software (version 27.0; SPSS) was used for all statistical analyses.

## 4. Conclusions

The present study demonstrated for the first time that an extract from the living plants (i.e., LR) of the brackish water angiosperm *R. maritima* possesses significant antioxidant activity, although its inhibitory activity against colon cancer cell growth was not too strong. Since *R. maritima* ecosystems have significant ecological importance and are protected by international conventions, the collection of living plants to obtain bioactive extracts is recommended providing that acceptable management plans without adverse impacts are applied to its ecosystems. Alternatively, the development of cultivation methods for these plants is proposed for their sustainable exploitation. Additionally, the present results suggested for the first time the use of *R. maritima* beach deposits (i.e., NR) as an alternative source of the plant’s bioactive compounds. This approach would be less environmentally invasive than the use of the living plants, while always considering sustainable management programs for seagrass beach deposits. Thus, the results showed that *R. maritima* NR extract retained a significant antioxidant potency compared to the LR extract. Surprisingly, the *R. maritima* NR extract exhibited much better inhibitory activity against colon cancer cell growth than the LR extract. Moreover, chemical composition analysis by HPLC showed that both *R. maritima* LR and NR extracts contained phenolics such as chicoric acid, quercetin-3-*O*-glucopyranoside, *p*-coumaric acid, *trans*-ferulic acid, sinapic acid, rutin hydrate, *trans*-cinnamic acid, and caftaric acid, all of which are known to possess antioxidant and/or anticancer activity.

Thus, the present findings indicate that *R. maritima* extracts from either LR or NR are a promising source of bioactive compounds that could be used for developing products beneficial to human health.

## Figures and Tables

**Table 1:** Phenolic compounds identified in LR and NR extracts from *Ruppia maritima* by UHPLC-DAD analysis.

Compounds	Rt (min)	LR Extract (mg/g d.w.)	NR Extract (mg/g d.w.)	LR Material ^b^ (mg/g d.w.)	NR Material ^b^ (mg/g d.w.)
Caftaric acid	4.56	T	T	T	T
Chicoric acid	12.42	41.09	45.40	11.50	4.68
Quercetin-3-*O*-glucopyranoside	22.53	10.22	10.74	2.86	1.10
Caffeic acid	ND	ND	ND	ND	ND
(-)-Epigallocatechin gallate	ND	ND	ND	ND	ND
*p*-Coumaric acid	11.87	9.14	10.76	2.55	1.10
*trans*-Ferulic acid	12.98	0.72 ^a^	0.82	0.20	0.08
Sinapic acid (3,5,Dimethy-4-hydroxycinnamic acid)	13.51	1.35	1.58	0.37	0.16
Rutin hydrate	16.82	5.26	6.00	1.47	0.61
*trans*-Cinnamic acid	20.66	0.17 ^a^	0.21 ^a^	0.04	0.02
Hesperidin	17.14	NQ	NQ	NQ	NQ
4′,5,7-Trihydroxyflavone	27.45	NQ	NQ	NQ	NQ
Total identified phenolics		67.95	75.51	19.02	7.76
Extraction yield (%)		28.00	10.30		

All numbers presented are over the LOQ. T: Traces, amounts below the detection limit of each compound (LOD). NQ: Not quantified (i.e., below the LOQ of each compound). Rt: Retention time. ND: compounds were not detected. (^a^): Values below the limit of quantification (LOQ) of each compound. (^b^): The values were evaluated by multiplying the amount of each phenolic compound (mg/g d.w. extract) by the percentage yield of each extract.

**Table 2:** Amount of individual phenolic compounds that could be produced from *Ruppia maritima* beach deposits according to the ‘case study’ in Monolimni Lagoon (Evros River Delta), along a shoreline of 100 m.

Phenolic Compounds	Amount (g)
Chicoric acid	263.88
Quercetin-3-*O*-glucopyranoside	62.36
*p*-Coumaric acid	62.48
*trans*-Ferulic acid	4.76
3,5-Dimethy-4-hydroxycinnamic acid (Sinapic acid)	9.17
Rutin hydrate	34.84
*trans*-Cinnamic acid	1.21
Total identified phenolics	438.71

## Data Availability

The data presented in this study are available on request from the corresponding authors.

## Supplementary Materials

The following supporting information can be downloaded at: https://www.mdpi.com/article/10.3390/molecules30132800/s1, File S1 ‘Materials and Methods in detail’.

## Abbreviations

The following abbreviations are used in this manuscript:

2,2-Azino-bis(3-ethylbenzthiazoline-6-sulfonic acid	ABTS^•+^
2,2-Diphenyl-picrylhydrazyl	DPPH
Ascorbic peroxidase	APX
Corrected total cell fluorescence	CTCF
Ethylenediaminetetraacetic acid	EDTA
Hydrogen peroxide	H_2_O_2_
Hydroxyl radical	OH^•^
Polyvinylpolypyrrolidone	PVPP
Reactive oxygen species	ROS
Reducing power	RP
*Ruppia maritima* beach deposits (or necromass)	NR
*Ruppia maritima* living plants	LR
Selectivity index	SI
Superoxide dismutase	SOD
Superoxide radical	O_2_^•−^

## References

[1] Davis W.P., Davis M.R., Flemer D.A., Bortone S.A. (2000). Observations on the Regrowth of Subaquatic Vegetation Following Transplantation: A Potential Method to Assess Environmental Health of Coastal Habitats. Seagrasses: Monitoring, Ecology, Physiology and Management.

[2] den Hartog C., Kuo J., Larkum A.W.D., Orth R.J., Duarte C.M. (2006). Taxonomy and Biogeography of Seagrasses. Seagrasses: Biology, Ecology and Conservation.

[3] Agulles M., Marbà N., Duarte C.M., Jordà G. (2024). Mediterranean Seagrasses Provide Essential Coastal Protection under Climate Change. Sci. Rep..

[4] Malea P., Kevrekidis T., Mogias A. (2004). Annual versus Perennial Growth Cycle in *Ruppia maritima* L.: Temporal Variation in Population Characteristics in Mediterranean Lagoons (Monolimni and Drana Lagoons, Northern Aegean Sea). Bot. Mar..

[5] Ito Y., Ohi-Toma T., Murata J., Tanaka N. (2010). Hybridization and Polyploidy of an Aquatic Plant, *Ruppia* (Ruppiaceae), Inferred from Plastid and Nuclear DNA Phylogenies. Am. J. Bot..

[6] Agostini S., Capiomont A., Marchand B., Pergent G. (2003). Distribution and Estimation of Basal Area Coverage of Subtidal Seagrass Meadows in a Mediterranean Coastal Lagoon. Estuar. Coast. Shelf Sci..

[7] Nonnis Marzano C., Scalera Liaci L., Fianchini A., Gravina F., Mercurio M., Corriero G. (2003). Distribution, Persistence and Change in the Macrobenthos of the Lagoon of Lesina (Apulia, Southern Adriatic Sea). Oceanol. Acta.

[8] Malea P., Kevrekidis T., Mogias A., Adamakis I.-D.S. (2014). Kinetics of Cadmium Accumulation and Occurrence of Dead Cells in Leaves of the Submerged Angiosperm *Ruppia maritima*. Bot. Mar..

[9] Christia C., Tziortzis I., Fyttis G., Kashta L., Papastergiadou E.A. (2011). Survey of the Benthic Aquatic Flora in Transitional Water Systems of Greece and Cyprus (Mediterranean Sea). Bot. Mar..

[10] Ragupathi Raja Kannan R., Arumugam R., Thangaradjou T., Anantharaman P. (2013). Phytochemical Constituents, Antioxidant Properties and *p*-Coumaric Acid Analysis in Some Seagrasses. Food Res. Int..

[11] Kim D.H., Mahomoodally M.F., Sadeer N.B., Seok P.G., Zengin G., Palaniveloo K., Khalil A.A., Rauf A., Rengasamy K.R. (2021). Nutritional and Bioactive Potential of Seagrasses: A Review. S. Afr. J. Bot..

[12] Zamani N.P., Rahman L., Rosada R.L., Tirtama W. (2021). Overview of Bioactivity Studies on Marine Natural Products. IOP Conf. Ser. Earth Environ. Sci..

[13] Kolsi R.B.A., Fakhfakh J., Krichen F., Jribi I., Chiarore A., Patti F.P., Blecker C., Allouche N., Belghith H., Belghith K. (2016). Structural Characterization and Functional Properties of Antihypertensive *Cymodocea nodosa* Sulfated Polysaccharide. Carbohydr. Polym..

[14] Kolsi R.B.A., Salah H.B., Saidi S.A., Allouche N., Belghith H., Belghith K. (2017). Evaluation of Nutritional Value, Characteristics, Functional Properties of *Cymodocea nodosa* and Its Benefits on Health Diseases. Lipids Health Dis..

[15] Ribas-Taberner M.D.M., Mir-Rossello P.M., Gil L., Sureda A., Capó X. (2025). Potential Use of Marine Plants as a Source of Bioactive Compounds. Molecules.

[16] Kevrekidou A., Assimopoulou A.N., Trachana V., Stagos D., Malea P. (2024). Antioxidant Activity, Inhibition of Intestinal Cancer Cell Growth and Polyphenolic Compounds of the Seagrass *Posidonia oceanica*’s Extracts from Living Plants and Beach Casts. Mar. Drugs.

[17] Hasle Enerstvedt K., Lundberg A., Jordheim M. (2017). Characterization of Polyphenolic Content in the Aquatic Plants *Ruppia cirrhosa* and *Ruppia maritima*—A Source of Nutritional Natural Products. Molecules.

[18] Malea P., Boubonari T., Kevrekidis T. (2008). Iron, Zinc, Copper, Lead and Cadmium Contents in *Ruppia maritima* from a Mediterranean Coastal Lagoon: Monthly Variation and Distribution in Different Plant Fractions. Bot. Mar..

[19] Malea P., Mylona Z., Kevrekidis T. (2019). Trace Elements in the Seagrass *Posidonia oceanica*: Compartmentation and Relationships with Seawater and Sediment Concentrations. Sci. Total Environ..

[20] Papathanasiou V., Kariofillidou G., Malea P., Orfanidis S. (2020). Effects of Air Exposure on Desiccation and Photosynthetic Performance of *Cymodocea nodosa* with and without Epiphytes and *Ulva rigida* in Comparison, under Laboratory Conditions. Mar. Environ. Res..

[21] Malea P., Charitonidou K., Sperdouli I., Mylona Z., Moustakas M. (2019). Zinc Uptake, Photosynthetic Efficiency and Oxidative Stress in the Seagrass *Cymodocea nodosa* Exposed to ZnO Nanoparticles. Materials.

[22] Adamakis I.-D.S., Malea P., Sperdouli I., Panteris E., Kokkinidi D., Moustakas M. (2021). Evaluation of the Spatiotemporal Effects of Bisphenol A on the Leaves of the Seagrass *Cymodocea nodosa*. J. Hazard. Mater..

[23] Liu J., Tang X., Wang Y., Zang Y., Zhou B. (2016). A Zostera Marina Manganese Superoxide Dismutase Gene Involved in the Responses to Temperature Stress. Gene.

[24] Malea P., Emmanouilidis A., Kevrekidis D.P., Moustakas M. (2022). Copper Uptake Kinetics and Toxicological Effects of Ionic Cu and CuO Nanoparticles on the Seaweed *Ulva rigida*. Environ Sci. Pollut. Res..

[25] Malea P., Kokkinidi D., Kevrekidou A., Adamakis I.-D.S. (2022). The Enzymatic and Non-Enzymatic Antioxidant System Response of the Seagrass *Cymodocea nodosa* to Bisphenol-A Toxicity. Int. J. Mol. Sci..

[26] Malea P., Dermentzis M., Patronia M.-M., Kevrekidis D.P., Kevrekidou A., Siopi V. (2024). Mechanism of Up-Regulated H_2_O_2_ BPA-Derived Production and Production of (Poly)Phenols by Two Seaweeds of the Genus *Ulva*. Environ Sci. Pollut. Res..

[27] Parthasarathi P., Umamaheswari A., Banupriya R., Elumalai S. (2021). Phytochemical Screening and in Vitro Anticancer Activity of Ethyl Acetate Fraction of Seagrass Halodule Uninervis from Mandapam Coastal Region, Rameswaram, Gulf of Mannar, India. Int. J. Pharm. Sci. Drug Res..

[28] Mohamed S.I.A., Elsayed G.H., El Shaffai A., Yahya S.M.M., Mettwally W.S.A. (2024). In-Vitro Study of Cytotoxic and Apoptotic Potential of *Thalassia hemprichii* (Ehren.) Asch. And *Enhalus acoroides* (L.f.) Royle against Human Breast Cancer Cell Line (MCF-7) with Correlation to Their Chemical Profile. BMC Complement. Med. Ther..

[29] Rubio-Portillo E., Martin-Cuadrado A.-B., Ramos-Esplá A.Á., Antón J. (2021). Metagenomics Unveils *Posidonia oceanica* “Banquettes” as a Potential Source of Novel Bioactive Compounds and Carbohydrate Active Enzymes (CAZymes). mSystems.

[30] Vasarri M., De Biasi A.M., Barletta E., Pretti C., Degl’Innocenti D. (2021). An Overview of New Insights into the Benefits of the Seagrass *Posidonia oceanica* for Human Health. Mar. Drugs.

[31] Harrison P.G. (1989). Detrital Processing in Seagrass Systems: A Review of Factors Affecting Decay Rates, Remineralization and Detritivory. Aquat. Bot..

[32] Achamlale S., Rezzonico B., Grignon-Dubois M. (2009). Evaluation of *Zostera detritus* as a Potential New Source of Zosteric Acid. J. Appl. Phycol..

[33] United Nations Environment Programme-World Conservation Monitoring Centre, UNEP-WCMC. https://unepwcmc.org/en/news/new-research-shows-which-mediterranean-coastal-communities-must-plan-now-for-seagrass-loss.

[34] Apostoloumi C., Malea P., Kevrekidis T. (2021). Principles and Concepts about Seagrasses: Towards a Sustainable Future for Seagrass Ecosystems. Mar. Pollut. Bull..

[35] de los Santos C.B., Scott A., Arias-Ortiz A., Jones B., Kennedy H., Mazarrasa I., McKenzie L., Nordlund L.M., de la Torre-Castro M., Unsworth R.K.F., UNEP (2020). Seagrass Ecosystem Services: Assessment and Scale of Benefits. Out of the Blue: The Value of Seagrasses to the Environment and to People.

[36] Tutar O., Marín-Guirao L., Ruiz J.M., Procaccini G. (2017). Antioxidant Response to Heat Stress in Seagrasses. A Gene Expression Study. Mar. Environ. Res..

[37] Bharathi N.P., Jayalakshmi M., Amudha P., Vanitha V. (2019). Phytochemical screening and in vitro antioxidant activity of the seagrass *Cymodocea serrulata*. Indian J. Geo-Mar. Sci..

[38] Messina C.M., Arena R., Manuguerra S., Pericot Y., Curcuraci E., Kerninon F., Renda G., Hellio C., Santulli A. (2021). Antioxidant Bioactivity of Extracts from Beach Cast Leaves of *Posidonia oceanica* (L.) *Delile*. Mar. Drugs.

[39] Krastanov A., Alexiera Z., Yernendzhiev H. (2013). Microbial degradation of phenol and phenolic derivatives. Eng. Life Sci..

[40] Agarry S.S.A., Durojaiye A.O., Solomon B. (2008). Microbial degradation of phenols: A review. Int. J. Environ. Pollut..

[41] Mejiaa S.Y., Rotini A., Lacasell F., Bookman R., Thaller M.C., Shem-Tov R., Winters G., Migliore L. (2016). Assessing the ecological status of seagrasses using morphology, biochemical descriptors and microbial community analyses.A study in *Halophila stipulacea* (Forsk.) Aschers meadows in thenorthern Red Sea. Ecol. Indic..

[42] Soto-Hernandez M., Palma-Tenango M., Garcia-Mateos R. (2017). Phenolic Compounds—Natural Sources, Importance and Applications.

[43] Kumar A., Sharma S., Kumar A.N. (2019). Microbes and Enzymes in Soil Health and Bioremediation.

[44] Ameen H.M., Jayadev A., Prasad G., Nair D.I. (2024). Seagrass Meadows: Prospective Candidates for Bioactive Molecules. Molecules.

[45] Adams J., Bate G. (1994). The tolerance to desiccation of the submerged macrophytes *Ruppia cirrhosa* (Petagna) Grande and Zostera capensis Setchell. J. Experim. Mar. Biol. Ecol..

[46] Shafer D.J., Sherman T.D., Wyllie-Echeverria S. (2007). Do desiccation tolerances control the vertical distribution of intertidal seagrasses?. Aquat. Bot..

[47] Lee J., Scagel C.F. (2013). Chicoric Acid: Chemistry, Distribution, and Production. Front. Chem..

[48] Ma J., Li M., Kalavagunta P.K., Li J., He Q., Zhang Y., Ahmad O., Yin H., Wang T., Shang J. (2018). Protective Effects of Chicoric Acid on H_2_O_2_-Induced Oxidative Injury in Hepatocytes and Larval Zebrafish Models. Biomed. Pharmacother..

[49] Li Z., Feng H., Han L., Ding L., Shen B., Tian Y., Zhao L., Jin M., Wang Q., Qin H. (2020). Chicoric Acid Ameliorate Inflammation and Oxidative Stress in Lipopolysaccharide and D -galactosamine Induced Acute Liver Injury. J. Cell Mol. Med..

[50] Nobela O., Renslow R.S., Thomas D.G., Colby S.M., Sitha S., Njobeh P.B., du Preez L., Tugizimana F., Madala N.E. (2018). Efficient Discrimination of Natural Stereoisomers of Chicoric Acid, an HIV-1 Integrase Inhibitor. J. Photochem. Photobiol. B Biol..

[51] Peng Y., Sun Q., Gao R., Park Y. (2019). AAK-2 and SKN-1 Are Involved in Chicoric-Acid-Induced Lifespan Extension in caenorhabditis Elegans. J. Agric. Food Chem..

[52] Kim M., Yoo G., Randy A., Kim H.S., Nho C.W. (2017). Chicoric Acid Attenuate a Nonalcoholic Steatohepatitis by Inhibiting Key Regulators of Lipid Metabolism, Fibrosis, Oxidation, and Inflammation in Mice with Methionine and Choline Deficiency. Mol. Nutr. Food Res..

[53] Yang M., Wu C., Zhang T., Shi L., Li J., Liang H., Lv X., Jing F., Qin L., Zhao T. (2022). Chicoric Acid: Natural Occurrence, Chemical Synthesis, Biosynthesis, and Their Bioactive Effects. Front Chem..

[54] DellaGreca M., Fiorentino A., Isidori M., Monaco P., Zarrelli A. (2000). Antialgal Ent-Labdane Diterpenes from *Ruppia maritima*. Phytochemistry.

[55] Haznedaroglu M.Z., Zeybek U. (2007). HPLC Determination of Chicoric Acid in Leaves of *Posidonia oceanica*. Pharm. Biol..

[56] Grignon-Dubois M., Rezzonico B. (2015). Phenolic Fingerprint of the Seagrass *Posidonia oceanica* from Four Locations in the Mediterranean Sea: First Evidence for the Large Predominance of Chicoric Acid. Bot. Mar..

[57] Grignon-Dubois M., Rezzonico B. (2013). The Economic Potential of Beach-Cast Seagrass—*Cymodocea nodosa*: A Promising Renewable Source of Chicoric Acid. Bot. Mar..

[58] Papenbrock J. (2012). Highlights in Seagrasses’ Phylogeny, Physiology, and Metabolism: What Makes Them Special?. ISRN Bot..

[59] Zeng W., Long X., Liu P., Xie X. (2023). The Interplay of Oncogenic Signaling, Oxidative Stress and Ferroptosis in Cancer. Int. J. Cancer.

[60] Tsukatani T., Ide S., Ono M., Matsumoto K. (2007). New tetrazolium method for phosphatase assay using ascorbic acid 2-phosphate as a substrate. Talanta.

[61] Apak R., Özyürek M., Güçlü K., Çapanoğlu E. (2016). Antioxidant Activity/Capacity Measurement. 2. Hydrogen Atom Transfer (HAT)-Based, Mixed-Mode (Electron Transfer (ET)/HAT), and Lipid Peroxidation Assays. J. Agric. Food Chem..

[62] Peng Y., Sun Q., Park Y. (2019). The Bioactive Effects of Chicoric Acid as a Functional Food Ingredient. J. Med. Food.

[63] Wang Y., Diao Z., Li J., Ren B., Zhu D., Liu Q., Liu Z., Liu X. (2017). Chicoric Acid Supplementation Ameliorates Cognitive Impairment Induced by Oxidative Stress via Promotion of Antioxidant Defense System. RSC Adv..

[64] Thygesen L., Thulin J., Mortensen A., Skibsted L.H., Molgaard P. (2007). Antioxidant Activity of Cichoric Acid and Alkamides from Echinacea Purpurea, Alone and in Combination. Food Chem..

[65] Nantitanon W. (2012). Comparison of Antioxidant Activity of Compounds Isolated from Guava Leaves and a Stability Study of the Most Active Compound. Drug. Discov. Ther..

[66] Kiliç I., Yeşiloğlu Y. (2013). Spectroscopic Studies on the Antioxidant Activity of *p*-Coumaric Acid. Spectrochim. Acta A Mol. Biomol. Spectrosc..

[67] Sheng K., Li Y., Wang Z., Hang K., Ye Z. (2021). p-Coumaric Acid Suppresses Reactive Oxygen Species-induced Senescence in Nucleus Pulposus Cells. Exp. Ther. Med..

[68] Nivetha S., Asha K.R.T., Srinivasan S., Murali R., Kanagalakshmi A. (2024). *p*-Coumaric Acid Pronounced Protective Effect against Potassium Bromate-induced Hepatic Damage in Swiss Albino Mice. Cell Biochem. Funct..

[69] Khelifi I., Hayouni E.A., Cazaux S., Ksouri R., Bouajila J. (2020). Evaluation of in Vitro Biological Activities: Antioxidant; Anti-Inflammatory; Anti-Cholinesterase; Anti- Xanthine Oxidase, Anti-Superoxyde Dismutase, Anti-Glucosidase and Cytotoxic of 19 Bioflavonoids. Cell Mol. Biol..

[70] Nićiforović N., Polak T., Makuc D., Poklar Ulrih N., Abramovič H. (2017). A Kinetic Approach in the Evaluation of Radical-Scavenging Efficiency of Sinapic Acid and Its Derivatives. Molecules.

[71] Akdemir F.N.E., Güler M.C., Eraslan E., Tanyeli A., Yildirim S. (2024). Assessment of Sinapic Acid’s Protective Effects against Ethanol-Induced Gastric Ulcers in Rats. Naunyn-Schmiedeberg’s Arch. Pharmacol..

[72] Ortasoz A.M., Ozdemir E., Taskıran A.S., Ozturk A. (2025). Sinapic Acid Alleviates Glutamate-Induced Excitotoxicity by Inhibiting Neuroinflammation and Endoplasmic Reticulum Stress Pathway in C6 Glioma Cells. Toxicol. Vitr..

[73] Akarsu S.A., İleritürk M., Küçükler S., Akaras N., Gür C., Kandemir F.M. (2024). Ameliorative Effects of Sinapic Acid against Vancomycin-Induced Testicular Oxidative Damage, Apoptosis, Inflammation, Testicular Histopathologic Disorders and Decreased Epididymal Sperm Quality. Reprod. Toxicol..

[74] Mehmood A., Soliman M.M., Almalki D.A., Alotaibi K.S., Youssef G.B.A., Althobaiti S. (2024). Ameliorative Impacts of Sinapic Acid against Mercuric Chloride-Induced Renal Toxicity: Role of Antioxidants and Inflammatory Cytokines. Toxicol. Res..

[75] Campoccia D., Ravaioli S., Santi S., Mariani V., Santarcangelo C., De Filippis A., Montanaro L., Arciola C.R., Daglia M. (2021). Exploring the anticancer effects of standardized extracts of poplar-type propolis: In vitro cytotoxicity toward cancer and normal cell lines. Biomed. Pharmacother..

[76] Mouna R., Broisat A., Ahmed A., Debiossat M., Boumendjel A., Ghezzi C., Kabouche Z. (2022). Antiproliferative Activity, Cell-Cycle Arrest, Apoptotic Induction and LC-HRMS/MS Analyses of Extracts from Two *Linum* Species. Pharm. Biol..

[77] Yoon J.-Y., Cho H.-S., Lee J.-J., Lee H.-J., Jun S.Y., Lee J.-H., Song H.-H., Choi S., Saloura V., Park C.G. (2016). Novel TRAIL Sensitizer *Taraxacum officinale* F.H. Wigg Enhances TRAIL-Induced Apoptosis in Huh7 Cells. Mol. Carcinog..

[78] Velusamy P., Muthusami S., Arumugam R. (2023). In Vitro Evaluation of P-Coumaric Acid and Naringin Combination in Human Epidermoid Carcinoma Cell Line (A431). Med. Oncol..

[79] Carmo-Martins J.I., Gonzatti M.B., Varela M.T., Sousa M.E.P., Costa L.V.S., Rodrigues E.G., Fernandes J.P.S., Keller A.C. (2023). Esterification of P-Coumaric Acid Improves the Control over Melanoma Cell Growth. Biomedicines.

[80] Radziejewska I., Supruniuk K., Tomczyk M., Izdebska W., Borzym-Kluczyk M., Bielawska A., Bielawski K., Galicka A. (2022). P-Coumaric Acid, Kaempferol, Astragalin and Tiliroside Influence the Expression of Glycoforms in AGS Gastric Cancer Cells. Int. J. Mol. Sci..

[81] Cui K., Wu H., Fan J., Zhang L., Li H., Guo H., Yang R., Li Z. (2022). The Mixture of Ferulic Acid and P-Coumaric Acid Suppresses Colorectal Cancer through lncRNA 495810/PKM2 Mediated Aerobic Glycolysis. Int. J. Mol. Sci..

[82] Oliva M.A., Castaldo S., Rotondo R., Staffieri S., Sanchez M., Arcella A. (2022). Inhibiting Effect of *p*-Coumaric Acid on U87MG Human Glioblastoma Cell Growth. J. Chemother..

[83] Tehami W., Nani A., Khan N.A., Hichami A. (2023). New Insights Into the Anticancer Effects of *p*-Coumaric Acid: Focus on Colorectal Cancer. Dose-Response.

[84] Eroğlu C., Avcı E., Vural H., Kurar E. (2018). Anticancer Mechanism of Sinapic Acid in PC-3 and LNCaP Human Prostate Cancer Cell Lines. Gene.

[85] Huang Z., Chen H., Tan P., Huang M., Shi H., Sun B., Cheng Y., Li T., Mou Z., Li Q. (2021). Sinapic Acid Inhibits Pancreatic Cancer Proliferation, Migration, and Invasion via Downregulation of the AKT/Gsk-3β Signal Pathway. Drug Dev. Res..

[86] Taştemur Ş., Hacısüleyman L., Karataş Ö., Yulak F., Ataseven H. (2023). Anticancer Activity of Sinapic Acid by Inducing Apoptosis in HT-29 Human Colon Cancer Cell Line. Can. J. Physiol. Pharmacol..

[87] Vasarri M., De Marchi L., Pretti C., Barletta E., Degl’Innocenti D. (2025). Antioxidant and Anti-Inflammatory Properties of Four Native Mediterranean Seagrasses: A Review of Bioactive Potential and Ecological Context. Mar. Drugs.

[88] Nieto G., Martínez-Zamora L., Peñalver R., Marín-Iniesta F., Taboada-Rodríguez A., López-Gómez A., Martínez-Hernández G.B. (2023). Applications of Plant Bioactive Compounds as Replacers of Synthetic Additives in the Food Industry. Foods.

[89] Cornara L., Pastorino G., Borghesi B., Salis A., Clericuzio M., Marchetti C., Damonte G., Burlando B. (2018). *Posidonia oceanica* (L.) Delile Ethanolic Extract Modulates Cell Activities with Skin Health Applications. Mar. Drugs.

[90] Ahmad M., Tahir M., Hong Z., Zia M.A., Rafeeq H., Ahmad M.S., Rehman S.U., Sun J. (2025). Plant and marine-derived natural products: Sustainable pathways for future drug discovery and therapeutic development. Front. Pharmacol..

[91] Jeong J.S., Kim J.W., Kim J.H., Chung E.H., Lee D.R., Choi B.K., Ko J.W., Kim T.W. (2024). Oral toxicity and genotoxicity assessment of standardized *Echinacea purpurea* (L.) extract and the pharmacokinetic profile of its active ingredient chicoric acid. Toxicol. Res..

[92] Ren J., Ren X., Ma L., Liu J., Yuan S., Wang G. (2024). Pharmacokinetics and antioxidant activity of dihydrocaffeic acid grafted chitosan nanomicelles loaded with chicoric acid in broilers. Poult. Sci..

[93] Petersen B., Egert S., Bosy-Westphal A., Müller M.J., Wolffram S., Hubbermann E.M., Rimbach G., Schwarz K. (2016). Bioavailability of quercetin in humans and the influence of food matrix comparing quercetin capsules and different apple sources. Food Res. Int..

[94] Konishi Y., Hitomi Y., Yoshioka E. (2004). Intestinal absorption of p-coumaric and gallic acids in rats after oral administration. J. Agric. Food Chem..

[95] Malik N., Dhiman P. (2022). New Approaches and Advancements in Drug Development from Phenolic P-coumaric *Acid*. Curr. Top Med. Chem..

[96] Posadino A.M., Cossu A., Giordo R., Zinellu A., Sotgia S., Vardeu A., Hoa P.T., Deiana L., Carru C., Pintus G. (2013). Coumaric acid induces mitochondrial damage and oxidative- mediated cell death of human endothelial cells. Cardiovasc. Toxicol..

[97] Seo Y.K., Kim S.J., Boo Y.C., Baek J.H., Lee S.H., Koh J.S. (2011). Effects of p-coumaric acid on erythema and pigmentation of human skin exposed to ultraviolet radiation. Clin. Exp. Dermatol..

[98] Bondonno N.P., Bondonno C.P., Rich L., Mas E., Shinde S., Ward N.C., Hodgson J.M., Croft K.D. (2016). Acute effects of quercetin-3-O-glucoside on endothelial function and blood pressure: A randomized dose-response study. Am. J. Clin. Nutr..

[99] ElGamal R., Song C., Rayan A.M., Liu C., Al-Rejaie S., ElMasry G. (2023). Thermal Degradation of Bioactive Compounds during Drying Process of Horticultural and Agronomic Products: A Comprehensive Overview. Agronomy.

[100] Bradford M.M. (1976). A Rapid and Sensitive Method for the Quantitation of Microgram Quantities of Protein Utilizing the Principle of Protein-Dye Binding. Anal. Biochem..

[101] Beyer W.F., Fridovich I. (1987). Assaying for Superoxide Dismutase Activity: Some Large Consequences of Minor Changes in Conditions. Anal. Biochem..

[102] Nakano Y., Asada K. (1981). Hydrogen peroxide is scavenged by ascorbate-specific peroxidase in spinach chloroplasts. Plant Cell Physiol..

[103] Goutzourelas N., Kevrekidis D.P., Barda S., Malea P., Trachana V., Savvidi S., Kevrekidou A., Assimopoulou A.N., Goutas A., Liu M. (2023). Antioxidant Activity and Inhibition of Liver Cancer Cells’ Growth of Extracts from 14 Marine Macroalgae Species of the Mediterranean Sea. Foods.

[104] “Quest Graph™ IC50 Calculator.” AAT Bioquest, Inc., 28 April 2025. https://www.aatbio.com/tools/ic50-calculator.

[105] Tsagias N., Koliakos I., Karagiannis V., Eleftheriadou M., Koliakos G.G. (2011). Isolation of mesenchymal stem cells using the total length of umbilical cord for transplantation purposes. Transfus. Med..

