# Supplementary material for: Comparison of Polyphenolic Content and Bioactivities Between Extracts from the Living Plants and Beach Deposits of the Submerged Brackish Water Angiosperm Ruppia maritima
Source: Molecules. 2025 Jun 29;30(13):2800. doi: 10.3390/molecules30132800 (PMC12250873; doi:10.3390/molecules30132800)
Supplement: Supplementary file 1 [file molecules-30-02800-s001.zip › molecules-3643522-supplementary.pdf]

SUPPLEMENTARY FILE S1

In the present Supplementary file, more details are given for some methods.

The number of the references and figures are referred to those of the main manuscript.

S1. Materials and Methods in Detail

S1.1. Chemical and Reagents

All chemicals and reagents used were of analytical grade and were obtained from Sigma-Aldrich (Burlington. MA, USA), unless otherwise stated.

S1.2. Imaging of Hydrogen Peroxide Production in Leaves from Living Plants (LR) and Beach Deposits (NR) of Ruppia maritima

The H_2O_2 production was assessed in *R. maritima* living leaves (i.e., LR) of different ages (juvenile, intermediate and adult leaves; n = 9) originating from 3 bundles, as well as in leaf material (n = 9 leaf pieces) from *R. maritima* beach deposits (i.e., NR) as described by Kevrekidou et al., [16]. Specifically, the plant materials were incubated in a solution of 25 μM 2, 7-dichlorofluorescein diacetate (DCF-DA) prepared in 10 mM dimethyl sulfoxide (DMSO) for 4 – 48 h. Subsequently, the samples were observed under a Zeiss AxioImager Z.2 fluorescence microscope (Carl Zeiss, Baden-Württemberg, Germany) with an epifluorescence system and a 488 nm filter. The cell fluorescence intensity was calculated with the Image J software version 1.54 software (U.S. National Institutes of Health, Bethesda, MD, USA). The corrected total cell fluorescence (CTCF) values were calculated according to the following equation:

$$\text{CTCF} = \text{integrated density} - (\text{area of selected cell} \times \text{mean fluorescence of background readings}) \quad \text{(S1)}$$

Their mean values were calculated from 81 measurements for LR leaves, namely, three regions per leaf x three segments (apex, middle, base) per leaf x 9 leaves, and from 27 measurements for NR material (i.e., three regions per leaf piece x 9 leaf pieces).

S1.3. Assessment of the Activity of the Antioxidant Enzymes SOD-like and APX-like and Protein Content in Leaves from Living Plants (LR) and Beach Deposits (NR) of Ruppia maritima

Three deep-freeze (−80 °C) leaf samples of both LR and NR materials were ground in liquid nitrogen. Each sample (100 mg wet weight) was treated in triplicate with 3 mL of 50 mM sodium phosphate buffer (pH 7.8), containing 0.1 mM of ethylenediaminetetraacetic acid (EDTA) and 2% *w*/*w* of polyvinylpolypyrrolidone (PVPP), then centrifuged at 16,500 × *g* for 30 min at 4 °C.

Then, the activity of the antioxidant enzymes superoxide dismutase (SOD)-like and ascorbate peroxidase (APX)-like, and the protein content of the samples were measured.

The protein content (mg/g) of the samples was measured, spectrophotometrically, according to the Bradford [100]. Specifically, 50 μL of each subsample was added to 5 mL of a solution containing 0.01% w/v Coomassie Brilliant Blue, 4.7% w/v ethanol and 8.5% w/v ortho-phosphoric acid. The optical absorbance was measured at 595 nm with a PharmaSpec UV-1700, Shimadzu spectrophotometer (Tokyo, Japan). The protein content was calculated based on a standard curve of bovine serum albumin.

SOD-like activity (U mg^{-1} protein) was measured according to the Beyer and Fridovich [101]. Specifically, 50 μL of each subsample (3 subsamples per sample) were added to 5 mL of a solution containing 50 mM potassium phosphate buffer (pH 7.8), 0.1 mM ethylenediaminetetraacetic acid (EDTA, Applichem), 0.025% v/v) 2-[4-(2,4,4-trimethylpentan-2-yl)phenoxy] ethanol, TritonX-100, 13 mM methionine, 0.075 mM nitro blue tetrazolium chloride (NBT), and 0.002 mM riboflavin. Then, SOD-like activity was measured at 560 nm using a PharmaSpec UV-1700, Shimadzu spectrophotometer (Tokyo, Japan), based on the inhibition rate of NBT reduction. The protein content of the samples was used to normalize the values.

APX-like activity (U mg^{-1} protein) was determined according to Nakano et al., [102]. Specifically, 50 μL of each subsample (3 subsamples per sample) were mixed with 5 mL of a reaction solution containing 50 mM potassium phosphate buffer, 0.1 mM ethylenediaminetetraacetic acid (EDTA) (pH 7.8), 0.5 mM ascorbic acid and 0.1 mM H_2O_2. APX-like activity was measured by monitoring the change in optical absorbance due to oxidation at 290 nm for 1 min using a PharmaSpec UV-1700, Shimadzu spectrophotometer (Tokyo, Japan). The protein content of the samples was used to normalize the values.

S1.4. Preparation of Ruppia maritima Extracts

The extracts from LR and NR samples were prepared as previously described [16]. In brief, to prepare the extract, the *R. maritima* materials were ground and soaked in a solution of 80% *v/v* methanol (1:30 dry weight sample to solvent volume). The solution was then subjected to sonication using an UP400S Hielscher sonicator (Teltow, Germany) for 20 min at 20 cycles and 70% amplitude. Afterward, the solution was placed in a shaker incubator (Innova® 40, New Brunswick Scientific; St Albans, UK) at 25 °C and 150 rpm for 48 h. Subsequently, the extract solution was filtered using a 0.45 μm Whatman filter paper. The solvent was then removed through rotary evaporation (IKA, Werke RV-06-ML; Staufen, Germany) at 30 °C and 150 rpm under reduced pressure, followed by freeze drying (CoolsafeTM, Scanvac, Allerod, Denmark) for 24 h, resulting in the extracts in powder form.

The weight of the dried powder was measured to determine the percentage yield of the extraction process, using the following equation:

$$\text{Extraction yield (\%)} = [\text{dry extract (g)}/\text{dry seaweed (g)}] \times 100 \quad \text{(S2)}$$

The extracts were kept at −20 °C until further use.

S1.5. Assessment of Phenolic Content in Ruppia maririma extracts

The phenolic compounds in LR and NR extracts were determined by UHPLC-DAD analysis as previously described [16]. Specifically, ECS05 UHPLC-DAD equipment (Prague, Czech Republic) consisting of a quaternary gradient pump (ECP2010H) and a gradient box with degasser (ECB2004) was employed coupled with a diode array detector (ECDA2800 UV-Vis PDA Detector). Chromatographic separation of the constituents was implemented on a Fortis Speed Core column (C18, 2.6 μm, 100 × 4.6 mm) (Cheshire, UK) at 25 °C. An aqueous mobile phase acidified with 0.1% formic acid (A) and a methanolic mobile phase (B) were utilized at a total flow rate of 1 mL/min. The elution gradient at t = 0 min was 90% A and remained constant for 5 min. While, at 8.5 min it was set to 72% A and at 30 min to 40% A, and then remained constant for 3 min. After each run (10 μL injection volume), equilibration was performed for 3 min at the initial conditions. Detection of the constituents was recorded at 280, 270, 328 and 318 nm. Data were processed by using Clarity Chromatography Software v8.2 (DataApex Ltd., Prague, Czech Republic). Identification and quantification of the individual phenolic compounds in all samples was based on the following mixture of standards: caffeic acid (purity 99%, J&K Scientific, Shanghai, China), *trans*-ferulic acid (purity 99%, Sigma Aldrich), *p*-coumaric acid (purity 98%, Sigma Aldrich), rutin hydrate (purity > 94%, Sigma Aldrich), *trans*-cinnamic acid (purity 99%, Sigma Aldrich), chicoric acid (purity > 98%, Glentham, Corsham, UK), caftaric acid (purity > 97%, Sigma-Aldrich), (-)-epigallocatechin gallate (purity 95%, Thermo Fisher Scientific, Waltham, NE, USA), 3,5-dimethoxy-4-hydroxycinnamic acid (sinapic acid; purity 98%, Sigma Aldrich), quercetin-3-O-glucopyranoside (purity > 99%, Extrasynthese, Genay Cedex, France), 4′,5,7-trihydroxyflavone (purity 97%, Thermo Fisher Scientific, Waltham, NE, USA) and hesperidin (purity 95%, Sigma Aldrich). Subsequently, the standards were diluted in methanol within a range of 0.78 – 200 mg/L to construct each calibration curve and were analyzed as mentioned above, at 280, 270, 328 and 318 nm. Analyses of the phenolic constituents were carried out in all extracts at 7 mg/mL in methanol. Calibration curves of each standard solution in methanol are depicted in Table S1.

Table S1. Calibration curve equations for phenolic compounds (at 280 nm).

Phenolic compounds	Equation	R^2	LOD (mg/L)	LOQ (mg/L)
**Caftaric acid**	y = 0,1897x + 0,7597	0,9996	8,08	24,47
**Caffeic acid**	y = 0,0425x - 0,0769	1,0000	2,04	6,19
**(-)-Epigallocatechin gallate**	y = 0,2767x + 1,3779	0,9999	4,11	12,45
***p*-Coumaric acid**	y = 0,0344x - 0,1693	0,9998	6,12	18,56
**Chicoric acid**	y = 0,1054x + 1,5392	0,9995	8,26	25,04
***trans*-ferulic acid**	y = 0,0439x - 0,1641	1,0000	1,7	5,16
**Sinapic acid (3,5-Dimethy-4-hydroxycinnamic acid)**	y = 0,0533x - 0,0896	0,9999	1,27	3,85
**Rutin hydrate**	y = 0,1517x + 0,1348	0,9999	1,18	3,58
***trans*-Cinnamic acid**	y = 0,0197x + 0,1115	0,9997	0,98	2,98
**Quercetin-3-*O*-glucopyranoside**	y = 0,1561x - 0,732	0,9996	6,65	20,16

Where y: concentration of phenolic compounds, x: area of each peak. LOD (Limit of Detection, mg/L). LOQ (Limit of Quantification, mg/L).

S1.6. Free Radical Scavenging Activity of Ruppia maritima Extracts

The DPPH• assay was performed as previously described [103]. In particular, the extracts were dissolved in double distilled water at a concentration of 10 mg/mL. Serial dilutions of the stock solution were made to obtain different extract concentrations. Then, 100 μL of each concentration was added to a methanolic DPPH• solution (100 μM), resulting in a final volume of 1 mL. After vortexing, the samples were incubated at room temperature in the dark for 20 min, and the absorbance was measured at 517 nm using a Perkin Elmer Lambda 25 UV/VIS spectrophotometer (Waltham, MA, USA). In each experiment, a negative control was included, which consisted of the tested sample alone in methanol. A control solution containing only DPPH• in methanol was also used.

The percentage of radical scavenging capacity (RSC) of the tested samples was evaluated according to the following formula:

$$RSC\ (\%) = [(A_{control} - A_{sample})/A_{control}] \times 100 \quad (S3)$$

where $A_{control}$ and A_{sample} are the absorbance values of the control and the sample, respectively. The IC_{50} value representing the concentration at which 50% of the DPPH• radical scavenging occurred, was calculated *using* a four parameter logistic regression model with the 'Quest Graph™ IC_{50} Calculator' (AAT Bioquest, Inc., Pleasanton, CA, USA) [104]. The experiment was performed in triplicate and repeated on at least three different occasions.

The ABTS•+ radical scavenging assay was carried out as described previously [103]. In summary, the ABTS•+ radical was produced by combining 500 μl of 2 mM ABTS, 50 μl of 30 μM hydrogen peroxide (H_2O_2), and 50 μl of 6 μM horseradish peroxidase (HRP) enzyme with 400 μL of distilled water. The mixture of reagents was thoroughly mixed and left to incubate at room temperature in the dark for 45 min. The extracts were dissolved in double

distilled water at a concentration of 10 mg/mL. Serial dilutions of the stock solution were made to obtain different extract concentrations. Subsequently, 10 μL of the different extract concentrations were added to the reaction mixture, and the absorbance was measured at a wavelength of 730 nm. In each experiment, the tested sample in distilled water containing $ABTS^{\bullet+}$ and H_2O_2 was used as a negative control. The $ABTS^{\bullet+}$ radical solution with 10 μL of H_2O was used as control. The percentage of RSC and the IC_{50} value of the tested extract were calculated as described above for the DPPH assay. The experiment was performed in triplicate and repeated on at least three different occasions.

The $^{\bullet}OH$ scavenging activity was determined as described previously [103]. In particular, different concentrations of extracts dissolved in distilled water (75 μL) were added to a mixture consisting of 450 μL sodium phosphate buffer (0.2 M, pH 7.4), 150 μL 2-deoxyribose (10 mM), 150 μL $FeSO_4$-EDTA (10 mM), 525 μL H_2O, and 150 μL H_2O_2 (10 mM). The samples were then incubated at 37 °C for 4 hours. Following the incubation, 750 μL trichloroacetic acid (TCA) (2.8%) and 750 μL 2-thiobarbituric acid (1%) were added, and the samples were incubated at 95 °C for 10 min. After cooling the samples on ice for 5 min, they were centrifuged at 3,000 rpm for 10 min at 25 °C. The absorbance was measured at 520 nm. Negative controls were included in each experiment, where samples without H_2O_2 were used. The samples without extract were used as controls. The RSC and the IC_{50} values for $^{\bullet}OH$ assay were evaluated as mentioned above for the $DPPH^{\bullet}$ radical. The experiment was performed in triplicate and repeated on at least three different occasions.

The $O_2^{\bullet-}$ scavenging activity of the extracts was evaluated as described previously [103]. In this assay, the extracts were dissolved in a Tris-HCl buffer (16 mM, pH 8.0) at various concentrations. The reaction mixture consisted of 125 μL of NBT_2^{+} (300 μM), 125 μL of NADH (468 μM), and 10 μL of extract (diluted in the buffer) added to 615 μL of Tris-HCl (16 mM; pH 8.0). The reaction was initiated by adding 125 μL of PMS (60 μM) to the mixture. The samples were then incubated in the dark for 5 min, and the absorbance was measured at 560 nm on a Perkin Elmer Lambda 25 UV/VIS spectrophotometer (Waltham, MA, USA). Each measurement included a blank containing 750 μL of Tri-HCl buffer, 125 μL of NBT, and 125 μL of NADH, as well as a control containing 625 μL of Tri-HCl buffer, 125 μL of NBT, 125 μL of NADH, and 125 μL of PMS. Negative controls were also included in each experiment, consisting of 740 μL of Tri-HCl buffer, 125 μL of NBT, 125 μL of NADH, and 10 μL of extract diluted in buffer. The RSC and the IC_{50} values for $O_2^{\bullet-}$ were determined in the same manner as mentioned above for the $DPPH^{\bullet}$ radical. The experiment was performed in triplicate and repeated on at least three different occasions.

In all free radical scavenging assays, ascorbic acid was used as a positive control.

S1.7. Reducing Power (RP) Activity of Ruppia maritima Extracts

Reducing power was determined spectrophotometrically as described previously [103]. In this assay, the extracts were dissolved in phosphate buffer (0.2 M, pH 6.6) at various concentrations. Two hundred and fifty microliters of the extract solution were combined with 250 μL of potassium ferricyanide (1% *w*/*v* in dH_2O) and incubated at 50 °C for 20 min. After incubation, the samples

were cooled on ice for 5 min. Subsequently, 250 μL of TCA (10% *w*/*v*) was added, and the samples were centrifuged (1,700 g, 10 min, 25 °C). Following centrifugation, 250 μL of distilled H_2O and 50 μL of ferric chloride (0.1% *w*/*v*) were added to the supernatant, and the samples were incubated at room temperature (RT) for 10 min. The absorbance was measured at 700 nm on a Perkin Elmer Lambda 25 UV/VIS spectrophotometer (Waltham, MA, USA). Each measurement included a blank containing 500 μL of phosphate buffer, 250 μL of TCA, 250 μL of dH_2O, and 50 μL of ferric chloride, as well as a control containing 250 μL of buffer, 250 μL of potassium ferricyanide, 250 μL of TCA, 250 μL of dH_2O, and 50 μL of ferric chloride. Negative controls were also included in each experiment, consisting of 250 μL of buffer, 250 μL of TCA, 250 μL of dH_2O, 50 μL of ferric chloride and 250 μL of extract diluted in buffer. The $RP_{0.5AU}$ value, indicating the extract concentration caused an absorbance of 0.5 at 700 nm, was *using* a four parameter logistic regression model with the 'Quest Graph™ IC_{50} Calculator' [104]. Ascorbic acid was used as a positive control. The experiment was performed in triplicate and repeated on at least three different occasions.

S1.8. XTT Assay for Assessing the Inhibitory Activity of Ruppia maritima Extracts against Cancer Cell Proliferation

The inhibition of cell proliferation by the tested extracts was assessed using the XTT assay kit (Roche, Germany), as described [103]. In particular, 1×10^4 LS174 colon cancer cells or normal MSCs were seeded into a 96-well plate in DMEM medium (DMEM, Gibko, UK). After 24 h of incubation, the cells were exposed to various concentrations of extracts in FBS-free DMEM medium for 24 h. Then, a mixture consisting of 50 μL of XTT-labeling reagent and 1 μL of electron coupling reagent was added to each well, followed by 4 h incubation. The absorbance was measured at 450 nm, with a reference wavelength of 690 nm, using a Perkin Elmer EnSpire Model 2300 Multilabel microplate reader (Waltham, MA, USA). Negative controls were cells cultured only in DMEM serum-free medium without the extract. Additionally, the absorbance of the extract alone in serum-free DMEM medium and XTT test solution was measured at 450 nm. The absorbance values obtained from the extract alone were subtracted from the absorbance values of the cells treated with the extract. In addition, the anticancer drug doxorubicin was used as positive control. The data were expressed as a percentage of inhibition using the following formula:

$$\text{Inhibition (\%)} = [(O.D._{control} - O.D._{sample})/O.D._{control}] \times 100 \quad \text{(S4)}$$

where $O.D._{control}$ and $O.D._{sample}$ indicated the optical density of the negative control and the tested extract, respectively. The IC_{50} value was calculated *using* a four parameter logistic regression model with the 'Quest Graph™ IC_{50} Calculator' [104]. The experiment was performed in triplicate and repeated on at least three different occasions.
